# UNDIAGNOSED DIABETES MELLITUS IN PATIENTS WITH HERPES ZOSTER

**DOI:** 10.4103/0019-5154.43211

**Published:** 2008

**Authors:** Mohammad Nassaji-Zavareh, Ramin Taheri, Raheb Ghorbani, Maryam Aminian

**Affiliations:** *From Department of Infectious Diseases, Semnan University of Medical Sciences, Semnan, Iran*; 1 *From Department of Dermatology, Semnan University of Medical Sciences, Semnan, Iran*; 2 *From Department of Social Medicine, Semnan University of Medical Sciences, Semnan, Iran*

**Keywords:** *Diabetes mellitus*, *fasting plasma glucose*, *herpes zoster*

## Abstract

**Background:**

Herpes Zoster (HZ) is reactivation of latent varicella-zoster virus that involves dermatomes. Aging and immunosupressed states are among the main risk factors. Some investigations showed that HZ is more common in diabetic patients than in normal population.

**Aim:**

To determine whether undiagnosed DM is more common in patients with HZ than in those without it.

**Materials and Methods:**

In this study 103 patients with HZ (cases) and 142 as control participated. They had no history of DM. Both groups were matched according to age, gender and family history of DM. Fasting plasma glucose was checked for all participants. DM was defined when the fasting plasma glucose was equal or more 126 mg/dl.

**Results:**

35.9% of patients with HZ and 19.7% of the control group had DM. There was significant association between HZ and undiagnosed DM (OR = 2.28, 95% CI: 1.28–4.06).

**Conclusion:**

Our findings indicate that the prevalence of undiagnosed DM is more common in HZ patients and supports the policy to investigate patients with HZ for the presence of undiagnosed DM.

## Introduction

Herpes zoster (HZ) is the reactivation of Varicella-Zoster virus (VZV) that becomes latent after primary infection within the dorsal root ganglia. It affects about 20% of the population mainly the elderly. The factors that are responsible for reactivation are not well known, but appear to be dependent on a balance between virus and host factors. During reactivation, VZV overwhelms immune control and spreads in the affected ganglia and sensory nerves to the skin.^1^

Immune dysfunction in certain diseases states is a potent trigger for HZ. Most commonly, advanced age, which acts as a surrogate for waning of cell-mediated immunity, is an important recognized risk factor.^2^ In patients with impaired immunity, both the incidence and severity of HZ are increased.^3^ This is seen in malignancies especially lymphoma, patients receiving immunosuppression therapy and HIV infection.^4^

Diabetes mellitus (DM) comprises a group of metabolic disorders that share the phenotype of hyperglycemia. The incidence of DM has increased in the past two decades. Individuals with DM have a greater frequency and severity of infections. Several rare infections are seen almost exclusively in diabetic population. The reasons for this include abnormality in cell mediated immunity and phagocyte functions associated with hyperglycemia.^5^

Undiagnosed DM especially type 2 is common, with an estimated lag of five to seven years between the onset of the disease and diagnosis. It is estimated that up to fifty percent of people are unaware of their disease. Incidence of undiagnosed DM increase with age. The size of the undiagnosed fraction of adults with diabetes is a major public health concern, heightened by the evidence that the latent stage is likely to be long, and that diabetes-related complications may develop.^6^

Early detection of DM and intervention improve long-term outcome, therefore it is important for the physicians to screen for DM in subjects who demonstrate major risk factors. Identification of such individuals may also be a worthwhile objective.^7^

The revised criteria for the diagnosis of DM emphasize the fasting plasma glucose (FPG) as a reliable and convenient test for diagnosis of DM in asymptomatic individuals.^5^

However, little is currently known concerning the clinical and socio-demographic characteristics of those individuals that they should constitute a target for the screening and management of impaired glucose level.

There are some reports indicating that HZ occurs more frequently in patients with diagnosed DM rather than normal population.^8^–^10^ Although suspected of being a marker of undiagnosed DM, information on this subject is scarce, and there are a few studies for evaluation of HZ as a marker of undiagnosed DM. If it could be proven that HZ is an indicator of DM, this could alert clinician to screening these patients for undiagnosed DM. This study was designed to evaluate the probable relationship between HZ and undiagnosed DM. On the other hand the objective was to answer this question; is undiagnosed DM more common in HZ patients than in those without it?

## Materials and Methods

In this case-control study, patients that visited in dermatology and infectious diseases clinics with HZ enrolled as cases. HZ was diagnosed for them by dermatologist and infectious specialist based on clinical findings. Patients that visited for other diseases in the same clinics, in the same duration and had no history of previous HZ enrolled as controls. In both groups, individuals with past history of DM or symptoms suggestive of DM (Polyuria, Polydypsia, Weight loss), those with known immunosuppressive disorders (HIV infections, chemotherapy, transplantation, neoplastic disorders) and those receiving drugs that increase blood glucose level (Corticosteroids, beta-blockers, thyroid hormones, thiazides, etc) were excluded. The presence of diagnosed diabetes was based on the patient's history and/or if they used hypoglycemic agents. Two groups were matched for age and gender. Family history of DM was recorded for both groups.

After informed consent, for each individual after twelve hours fasting blood sample was taken for measurement of FPG using standard laboratory test. For overcoming stress-induced hyperglycemia, FPG of cases was measured after healing of vesicular eruption and subsiding pain. If control subjects had any acute disease, their FPG was measured after subsiding problem. Subjects were considered as having undiagnosed diabetes when FPG level was equal or over 126 mg/100 ml.^11^

Kolmogrov-Smirnov, Independent samples t, *Chi-square*, Fisher exact tests, Odds Ratio (OR) and 95% Confidence Interval (CI) were used for the statistical evaluation of the data. *P*-value less than 0.05 were considered statistically significant. Statistical analysis was performed by SPSS version 9.0 for windows.

## Results

103 patients with HZ and 142 patients without HZ fulfilled inclusion criteria. Mean (±SD) age of cases was 51.9 (±16.6) and for controls was 50.2 (±14.3), that difference was not significant (*P* = 0.407). The case group included 53 (51.5%) and controls 64 (45.1%) female (*P* = 0.233). Family history of DM was positive in 1% of HZ patients and 1.4% of those without HZ (Table 1).

**Table 1:** Characteristics of two groups

Factor	Study group				*P*-value
		
	Herpes zoster *n* %		Control *n* %		
Age					
<50.0	48	46.6	71	50	NS*
≥50.0	55	53.4	71	50	
Gender					
Female	53	51.5	64	45.1	NS
Male	50	48.5	78	54.9	
Family history of DM					
+	1	1.0	2	1.4	NS
-	102	99.0	140	98.6	

* NS: Not significant

35.9% of patients with HZ and 19.7% of controls had FPG ≥ 126. There was a significant association between HZ and undiagnosed DM (OR = 2.28, CI 95%: 1.28-4.06)

## Discussion

Increased incidence and severity of HZ have been approved in some immunosuppressed states. In a study, 28.4% of 67 patients with HZ were immunocompromised. Nine patients had malignancy and 13 patients had been on cytotoxic and/or steroid therapy.^12^

In Donahue *et al*, study, from 1075 cases with HZ, HIV infection was documented in 5% and cancer in 6%.^13^

DM can cause abnormalities in various parts of immunity system and can increase risk and severity of infections. Our study showed significant association between HZ and undiagnosed DM. The estimated odds ratio of HZ group was 2.28 in relation to control group. In McCulloch study 12.7% 0f 1017 diabetic patients had past history of HZ and 61% of these patients developed HZ before the onset of DM.^14^ In accordance with our study, Neu and Rodiek detected disorder of glucose utilization in 16 of 28 HZ patients.^8^

In another study it was found that among 140 patients with HZ, 13.5% of patients had DM, which is significantly higher than general incidence of 2%. When patients over 50 years old were considered separately, the incidence goes up to 17%.^15^ In Cerny study 12 patients with recurrent HZ were evaluated. Three of the patients had DM.^16^ In 31 cases of HZ with neurological complications; smoking with diabetes was the putative risk factors in 53%.^9^

In contrast to our finding in a prospective study on 590 patients with HZ, the clinical spectrum of the disease was not different from general population. They concluded that HZ is not a risk factor for DM and diabetes was not a risk factor for HZ.^17^

Our study has several limitations. First, the number of patients is relatively small.

Secondly, our patients were from same province. Study in other areas is recommended. Based on our finding we conclude that undiagnosed DM is more common in HZ patients than in patients without this disease. It may be related to deterioration of immunity. We therefore, recommend that HZ can be considered as a criterion for screening of undiagnosed DM. More research is needed to test this hypothesis.
